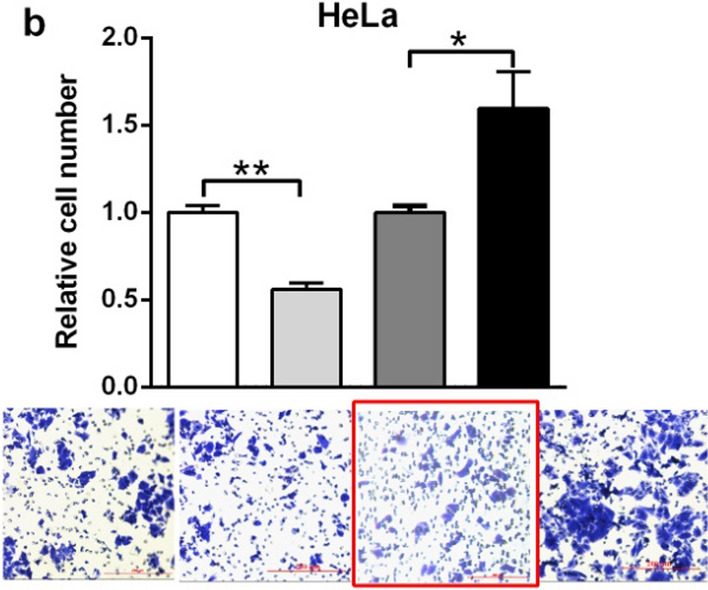# Correction to: RETRACTED ARTICLE: miR-484 suppresses proliferation and epithelial–mesenchymal transition by targeting ZEB1 and SMAD2 in cervical cancer cells

**DOI:** 10.1186/s12935-020-01375-9

**Published:** 2020-07-17

**Authors:** Yang Hu, Hong Xie, Yankun Liu, Weiying Liu, Min Liu, Hua Tang

**Affiliations:** Tianjin Life Science Research Center and Department of Pathogen Biology, School of Basic Medical Sciences, Tianjin Medical University, 22 Qi‑Xiang‑Tai Road, Tianjin, 300070 China

## **Correction to:** Cancer Cell Int (2017) 17:36 10.1186/s12935-017-0407-9

Following publication of the original article [1], the authors notified us the transwell invasion image for ASO-NC of HeLa cell in Fig. 3b was wrongly placed by mistake during the preparation of the figure. The corrected version of Fig. 3b is provided below (in the red box). This correction does not change the conclusions of this paper.